# Sedentary Behaviors and Physical Activity of Italian Undergraduate Students during Lockdown at the Time of CoViD−19 Pandemic

**DOI:** 10.3390/ijerph17176171

**Published:** 2020-08-25

**Authors:** Francesca Gallè, Elita Anna Sabella, Stefano Ferracuti, Osvalda De Giglio, Giuseppina Caggiano, Carmela Protano, Federica Valeriani, Eduardo Alfonso Parisi, Giuliana Valerio, Giorgio Liguori, Maria Teresa Montagna, Vincenzo Romano Spica, Giovanna Da Molin, Giovanni Battista Orsi, Christian Napoli

**Affiliations:** 1 Department of Movement Sciences and Wellbeing, University of Naples “Parthenope”, Via Medina n. 40, 80133 Naples, Italy; giuliana.valerio@uniparthenope.it (G.V.); giorgio.liguori@uniparthenope.it (G.L.); 2 Inter-University Research Centre “Population, Environment and Health”, University of Bari Aldo Moro, Piazza Umberto I, 1, 70121 Bari, Italy; elita.sabella@uniba.it (E.A.S.); giovanna.damolin@uniba.it (G.D.M.); 3 Department of Human Neurosciences, “Sapienza” University of Rome, Piazzale Aldo Moro 5, 00185 Rome, Italy; stefano.ferracuti@uniroma1.it; 4 Department of Biomedical Science and Human Oncology, University of Bari Aldo Moro, Piazza G. Cesare 11, 70124 Bari, Italy; osvalda.degiglio@uniba.it (O.D.G.); giuseppina.caggiano@uniba.it (G.C.); mariateresa.montagna@uniba.it (M.T.M.); 5 Department of Public Health and Infectious Diseases, “Sapienza” University of Rome, Piazzale Aldo Moro 5, 00185 Rome, Italy; carmela.protano@uniroma1.it (C.P.); giovanni.orsi@uniroma1.it (G.B.O.); 6 Department of Movement, Human and Health Sciences, University of Rome “Foro Italico”, Piazza Lauro De Bosis 15, 00135 Rome, Italy; federica.valeriani@uniroma4.it (F.V.); vincenzo.romanospica@uniroma4.it (V.R.S.); 7 Department of Medical Surgical Sciences and Translational Medicine, “Sapienza” University of Rome, Via di Grottarossa 1035/1039, 00189 Rome, Italy; eduardoparisiroma@gmail.com (E.A.P.); christian.napoli@uniroma1.it (C.N.)

**Keywords:** CoViD-19, lockdown, sedentary behaviors, physical activity, undergraduates

## Abstract

*Background*: From March to May 2020, lockdown measures were adopted in Italy to contain the epidemic of the novel Coronavirus. People were forced to restrict their movement and social contacts, therefore having a higher risk of inactivity. This study, carried out among Italian undergraduates, explored their sedentary and physical activities (PA) during the lockdown with respect to their previous habits. *Methods*: An electronic questionnaire was administered once to students attending three Italian universities after the end of lockdown. *Results*: A total of 1430 students (mean age 22.9 ± 3.5 years, 65.5% females) completed the questionnaire. All the sedentary behaviors increased significantly, and all the physical activities decreased significantly during the lockdown. Time spent using electronic devices showed the highest increase (+52.4 min/day), and walking the greatest decrease (−365.5 min/week). Being younger than 22 years old, female, and previously active, attending the universities of Naples and Rome and having at least one graduate parent were associated with the achievement of recommended levels of PA even during the lockdown. *Conclusions*: This study highlights the reduction of PA among Italian undergraduates in the course of home-confinement due to the CoViD-19 pandemic. The practice of adequate PA during the lockdown was mainly associated with the previous adoption of an active lifestyle. Promoting active lifestyles during the non-pandemic period may have had positive effects also in case of lockdown.

## 1. Introduction

Since its onset, the pandemic caused by the novel coronavirus (2019-nCoV) has posed important health challenges to populations worldwide, taking into account also specific epidemiological contexts [1,2,3]. In addition to the morbidity and mortality directly linked to the Coronavirus-19 Disease (CoViD-19), many governments adopted response measures that also had important physical and psychological implications for individuals [1,2,4,5,6]. In fact, in order to control the spread of the disease and reduce the impact on health systems, people were recommended or imposed to avoid social interactions and stay at home [2]. In Italy, where the CoViD-19 epidemic started in February 2020, lockdown measures were applied first in the northern regions and subsequently in the whole Italian territory [7,8]. People were allowed to move only for work or health reasons or to buy essentials. Therefore, the great part of the Italian population was forced to live in home-confinement for weeks, increasing the risk of reducing their physical activity (PA) and of adopting sedentary behaviors, intended as those behaviors performed in a sitting or reclining posture requiring an energy expenditure ≤1.5 Metabolic Equivalents (METs) [7].

Inactivity and sedentary behavior have numerous negative effects on human health at muscular, cardiovascular, metabolic and endocrine levels, also affecting psychological wellbeing [6,9,10]. These effects are mediated by mechanisms such as the development of muscle atrophy, the reduction of muscle insulin sensitivity and aerobic capacity and changes in body composition, which may occur even after limited periods of inactivity [4,5]. University students in particular are exposed to unhealthy sedentary and dietary habits also in the non-pandemic period, especially if living away from home [11].

With the adoption of social distancing measures such as the closure of gyms, sports facilities and parks, the achievement of the WHO (World Health Organization) recommended PA levels (at least 150 min of moderate-to-vigorous PA or 75 min of intensive PA per week, or a combination of both in adults) [10] became challenging. In this scenario, the use of home-based exercise programs may be considered relevant to counteract the deleterious effects of increased inactivity [5]. Thus, several recommendations have been issued by international and national institutions during the CoViD-19 epidemic to highlight the importance of staying physically active during quarantine and some of these reported examples of home-based exercises [12,13,14].

Nevertheless, in the course of the present epidemic, a higher knowledge regarding CoViD-19 was found to be associated with a lower likelihood of negative attitudes and of potentially dangerous practices in the Chinese population [15]. Therefore, also in Italy, a multicenter study was launched in order to investigate the level of knowledge about the disease and the lifestyle adopted by Italian undergraduates during the current epidemic [16]. The first and preliminary part of the study testified to an acceptable level of undergraduate student knowledge regarding the epidemic and the control measures adopted. As for lifestyles, the majority of the students did not modify their diet and smoking habits but reported a significant decrease of PA level. Nevertheless, during this first part of the study, lifestyles and their determinants were not investigated in depth, in order to avoid an excessive length of the questionnaire [16]. Therefore, we carried out a second phase of the study to investigate more fully criticisms related to inactivity induced by movement restriction measures in Italy, in the same population as the first phase. The aims of the second study phase were: (i) to evaluate the sedentary activities and PA levels the students assumed during home-confinement with respect to their previous habits, and (ii) to investigate the possible determinants of enduring PA during the lockdown.

## 2. Materials and Methods

In order to assess the behaviors of students during the lockdown in comparison with their previous habits, a cross-sectional study investigating their PA-related habits before and during the lockdown was designed. An electronic questionnaire proposed during academic lessons was used to collect these information.

### 2.1. Setting and Participants

The second phase of the “Survey on knowledge and behaviors of undergraduates during the EPIdemic of Coronavirus-19” (EPICO study), used an investigation tool named “UnLock—Survey on UNdergraduates behaviors during the LOCKdown”, and took place across the last three weeks of May 2020. The study involved students from three Italian universities: Sapienza University of Rome, Parthenope University of Naples and Aldo Moro University of Bari. On the basis of the emergency measures established by the Italian Government, no classroom lessons were allowed in that period and the three enrolled universities provided their lessons via internet [7]. Students attending web courses were invited, during lessons, to voluntarily participate in the study by filling in a web-based questionnaire.

The estimated total population of the three universities was 166,703 students. Therefore, a sample of at least 384 enrolled individuals would have been required to investigate the selected variables in the student population examined, assuming a response proportion of 50%, a 95% confidence level and a 5% margin error, as reported in previous studies [16,17].

The study did not include any experiments involving human or biological human samples, nor any research on identifiable human data. Participants expressed their informed consent prior to filling in the questionnaire. Ethical approval was obtained by the Scientific committee of the Inter Universities Research Centre “Population, environment and health” (CIRPAS_1603_2020 of the 1 April 2020) in conformity with the principles embodied in the World Medical Association Declaration of Helsinki and with the general data protection regulations.

### 2.2. Questionnaire

A web-based questionnaire in the Italian language was created and administered via Google modules. The first section of the questionnaire included questions regarding socio-demographic features of participants (gender, age, nationality, university, parents’ educational level, residential status) and specific conditions during lockdown (living with own family or not if off-site student, have been in quarantine or not due to personal or contacts’ exposure to 2019-n-CoV). The design of this section was based on previous studies performed with the same student population [11,16] and revised by a panel of experts composed of one demographer and one epidemiologist.

In order to assess undergraduates’ weekly time spent in sedentary behaviors and levels of habitual PA, questions from the Adult Sedentary Behaviour Questionnaire (ASBQ) and from the International Physical Activity Questionnaire (IPAQ) were included in the questionnaire [18,19]. The ASBQ is a seven-items validated questionnaire aimed at estimating total and specific sedentary behaviors related to work, transportation or leisure time [18,20,21,22]. It includes questions investigating the time (expressed in hours and minutes per working and non-working days) an individual spent during a typical week in work/studying activities, motorized sitting for transportation, eating meals, television viewing, performing activities on computers, tablets or mobile phones, or other leisure time activities [18]. Considering that during the lockdown all activities outside home were not allowed, we did not ask students to distinguish between working and non-working days, and the question regarding transportation was avoided. A specific question investigating the amount of sleeping/napping time extracted from the Last 7-day Sedentary Time Questionnaire (SIT-Q-7d) was also posed to participants [22]. Habitual PA levels were investigated through the short version of the IPAQ, which allowed assessment of the time (expressed in total number of minutes per week) spent by undergraduates in vigorous or moderate PA and walking [19]. According to IPAQ definitions, vigorous PA was indicated as an exercise which requires intense work and leads to abundant sweating, while moderate PA was indicated as modest work with moderate perspiration. Each question investigating sedentary behaviors and PA was posed twice: one referred to the lockdown and one to the pre-pandemic period.

Three additional questions regarding habits of doing housework, going grocery shopping and walking pets as indicators of PA during lockdown were also asked. The possible answers for these last questions were *yes* or *not*.

The design of this section was evaluated and modified by a panel of experts composed of one public health professional, one movement science expert and one psychologist. The questionnaire had been previously tested in a pilot-study (data not published).

The measure of questions’ comprehensibility was evaluated in a group of 95 undergraduate students who were not included in the larger study sample, as previously performed [16]. The 95 students were asked to assign a rating to each question on a 7-point scale (Replying to the question: Does the following sentence make sense to you? 1: not meaningful; 7: very meaningful); a mean score >5 per question was considered as the cut off for acceptability. For this purpose, the original questionnaire was modified: aside from the questions belonging to the standard questionnaire (SQQ), 15 additional questions (AQ) reporting grammatical and semantic errors were included, in order to guarantee answer variability. SQQ reported a mean score for each question ≥5.5; AQ reported a mean score for each question ≤3. These data confirmed that the content of the questionnaire was clear to the readers. The 95 students were also asked to reply to the final version of the questionnaire, in order to evaluate the reliability index [23,24].

### 2.3. Statistical Analyses

A descriptive analysis was performed considering socio-demographic characteristics, habits and behaviors reported by participants.

Continuous normally and non-normally distributed variables were expressed as mean values ± standard deviation (SD) or median values ± interquartile range (IQR), respectively. The Wilcoxon Signed Ranks Test was used to compare median values of variables between the two periods considered (indicated as *T*_0_ = before lockdown and *T*_1_ = lockdown). The Pearson’s *r* value was calculated as a measure of effect size for these comparisons (small 0.10−<0.30, medium 0.30−<0.50, large ≥0.50) [25].

A logistic regression analysis was performed considering the achievement of recommended PA levels as the outcome. The dependent variable was built attributing the value 0 to the participants who did not declare that they had fulfilled 150 min/week of moderate-to-vigorous PA, and 1 to the others. Age (classified as lower/equal or higher than the median value), gender, university, parents’ educational level (classified as having at least one graduate parent or not), BMI (classified as under/normal weight and overweight/obese) and achieving recommended PA levels before the lockdown were assumed as independent variables. Results were expressed with Odds Ratio values with corresponding 95% Confidence Intervals.

Data were analyzed using IBM SPSS version 26 for Windows (IBM Corp., Armonk, NY, USA).

## 3. Results

From the whole student population of the three Universities (*n* = 166,703), a total of 1430 students completed the questionnaire.

Cronbach’s alpha (internal consistency coefficient) values for the questionnaire used in the study were 0.73 and 0.79 [23,24], for the pilot and original study, respectively. The values achieved showed a satisfactory level of reliability [23,24].

The socio-demographic characteristics of the sample are reported in Table 1.

The sample was mainly composed of female, Italian and resident on-site students. The majority of the students reported a high-school educational level for their parents. The majority of the off-site students spent the lockdown with their own family. Only about 2% of the total sample underwent a quarantine for having had contacts with infected people. As for the type of PA, the majority of the respondents reported housework, while about half of the sample reported grocery shopping and only 24.3% walked pets during the lockdown.

Table 2 shows the differences registered between *T*_0_ and *T*_1_ in BMI, total sedentary time, sleeping/napping time, and total PA.

It is possible to observe a significant increase of total time spent in sedentary behaviors and sleeping/napping during the lockdown and a significant decrease of total PA, with corresponding medium-to-large effect size values, while BMI did not change between *T*_0_ and *T*_1_.

**Figure 1:**
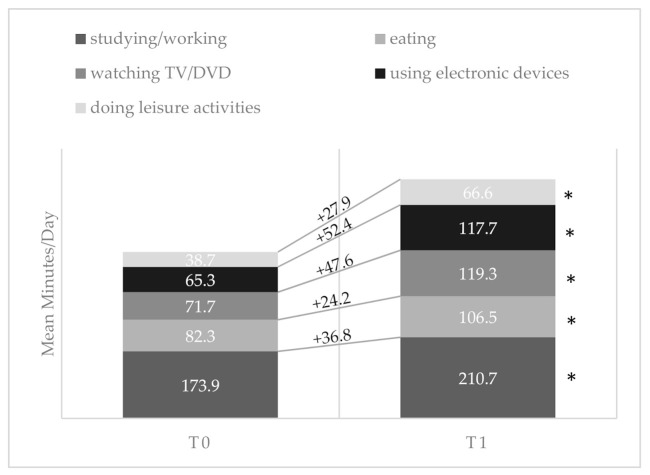
Mean time spent by participants in specific sedentary behaviors before (*T*_0_) and during (*T*_1_) the lockdown with corresponding change values. * *p* < 0.05.

**Figure 2:**
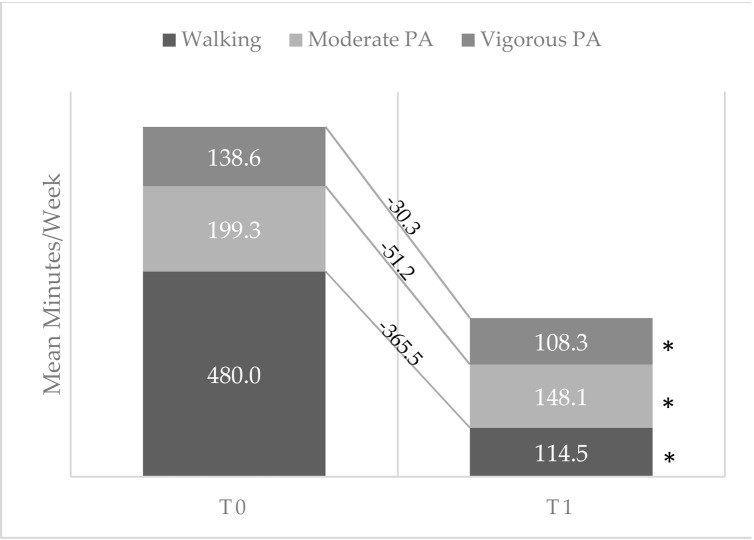
Mean time spent by participants in walking, moderate and vigorous Physical Activity (PA) before (*T*_0_) and during (*T*_1_) the lockdown with corresponding change values. * *p* < 0.05.

Figure 1 and Figure 2 show the changes in mean values of sedentary time and PA components between the two times considered.

All the sedentary behaviors showed a significant increase, and all the components of PA showed a significant reduction. The highest increase among sedentary components was registered for performing activities on electronic devices (+52.4 min/day), while walking was the PA component which had the greatest decrease (−365.5 min/week).

A total of 639 participants (44.7%) remained sufficiently active during the lockdown. The results of the linear regression analysis performed considering the achievement of recommended PA levels during lockdown as the outcome show that being younger than 22 years old, female, and previously active, attending the universities of Naples and Rome, and having at least one graduate parent were associated with the achievement of recommended levels of PA during the lockdown (Supplementary Table S1).

## 4. Discussion

This study aimed to assess the changes in sedentary behaviors and PA among Italian undergraduates during the lockdown related to the CoViD-19 pandemic. The results testify a significant increase of time spent in sedentary activities and a consistent decrease in PA during home-confinement.

This is in line with our previous investigation, which showed that during home-confinement 48.6% of the participants from the same student population decreased their PA levels, and with other research performed with college students and adults from the United States of America and France, respectively [16,26,27]. Indeed, another study carried out in Belgium by Constandt et al. reported a general increase of exercise, even with a concomitant increase of sedentary behaviors, in the Belgian adult population during the CoViD-19 lockdown [28]. These contrasting results were due to the fact that people who were previously under-active reported exercising more during the lockdown; while, among individuals who were already highly active before the pandemic, those above 55 years old, those with low education, those who used to exercise with friends or in a sport club, and those who were not using online tools to exercise, reported exercising less during home-confinement. Similarly, Cheval et al. reported an increase in leisure-related PA as well as in leisure-related sedentary activity in people from France and Switzerland [29].

In our previous study, 16% of the students reported the maintenance of their habitual PA levels during the lockdown and 21.3% increased their PA with respect to the past, suggesting that a part of this population was able to maintain an active lifestyle [16]. In the present study, despite a general consistent reduction in PA, a part of the sample succeeded in achieving sufficient PA levels during home-confinement. The regression analysis showed that this was associated with age, parents’ education and previous PA levels, according to the literature [28,30,31], and with female gender and university attended. Contrary to these findings, in the study by Constandt et al. females represented the majority of the under-active people subsample during lockdown [28]. However, it should be noted that this examined the whole adult population, with an age ranging from 18 to 74 years, while our investigation was focused on young adults. Furthermore, in a previous lifestyle analysis involving undergraduates from Bari and Naples, we found that PA levels were significantly different between the two universities. It is possible that even geographical reasons may have played a role in the determination of PA maintenance during lockdown [11]. Moreover, it should be noted that the two phases of the study were performed in different periods of the lockdown. The first one was carried out in the very early phase of the lockdown, the second at the end of the restriction period.

Surprisingly, the general reduction in PA registered in this study was not accompanied by a corresponding increase in BMI. However, it should be considered that in the previous investigation the majority of the sample declared that they did not change, or had improved, their diet. Therefore, it is possible that the negative effects of inactivity on BMI might have been balanced by healthier dietary habits.

Performing activities on electronic devices was the type of sedentary behavior which showed the highest increase. The investigation carried out by Cellini et al. among Italian adults including university students reported an increase of digital media usage before going to bed during the lockdown, although this change did not affect sleep habits [32]. Instead, our sample reported a significant increase in total bedtime.

Walking was the main type of PA reduced during the lockdown. This is explainable by the restriction of outdoor movements. In fact, grocery shopping and walking pets, the only activities allowed during the lockdown, were reported only by a half and a quarter of the sample, respectively. Furthermore, during the pandemic lessons were offered online in Italy, and the students did not attend university locations. This inevitably contributed to the reduction of their walking time. In contrast with our results, an increase in leisure-related walking activity has been reported for adults living in France and Switzerland [29].

This study has some limitations. First of all, it aimed to investigate the lifestyle of a sample of undergraduate students from three Italian universities, who represent a specific population group, and individuals of the same age group not attending universities were not enrolled as control. Therefore, the findings cannot be extended to the whole population of young adults in Italy. Moreover, female students were more represented in the sample: this is probably due to the different levels of compliance to such an investigation of the two genders, as reported in similar studies [15,16,17]. The number of students enrolled is also lower than the first study, probably due to the greater length and difficulty in fulfilling the questionnaire. Furthermore, this investigation was purposely focused on sedentary behaviors and PA and the questionnaire did not include questions regarding dietary habits, which could have given other important information regarding the lockdown. We also missed investigating in depth exercise practiced before the lockdown: this information might have been useful in highlighting differences in PA maintenance among sports professionals, amateur, and sedentary individuals. Finally, considering that all the information was self-reported by participants, it is possible that they under- or overestimated their weight and the time spent in sedentary behavior or PA. The use of objective measuring tools such as accelerometers or pedometers might have allowed more accurate quantification of time spent in sedentary activities, PA and its components. However, the use of a web-based questionnaire allowed to obtain a wide sample size. Moreover, the study was carried out immediately after the end of the lockdown, in order to improve the reliability of the students’ recall.

The information collected in this investigation highlights the need for interventions of PA promotion targeted at the examined population. During a lockdown, the web may be a useful resource to transmit health promotion messages, and indeed it was employed by international and national institutions [12,13,14]. However, the literature shows that web-based interventions are less effective in promoting PA among university students than in-person interventions aimed at addressing their motivation and skills related to adopting an active lifestyle [33]. Considering that previously adopted behaviors seem to be associated with those adopted during the lockdown, it could be opportune to promote PA among undergraduates outside emergency situations such as the CoViD-19 epidemic.

## 5. Conclusions

Notwithstanding its limitations, this investigation offers a contribution to the characterization of the collateral effects of the lockdown on people’s behaviors.

The findings of this study testify to a consistent reduction of PA in Italian university students during the lockdown related to the CoViD-19 pandemic. However, being previously active was associated with the fulfillment of recommended levels of PA even during home-confinement. The promotion of an active lifestyle during a pandemic is essential to counteract the possible negative effects of inactivity on people’s health. At the same time, promoting PA during the non-pandemic period may also have positive effects in case of lockdown.

## Figures and Tables

**Table 1:** Characteristics of the sample.

Variable	Participants *n =* 1430
Age *(years)*	
*Mean ± SD*	22.9 ± 3.5
*Median value*	22
Gender	
*n (%)*	
Male	494 (34.5)
Female	936 (65.5)
Nationality	
*n (%)*	
Italian	1345 (94.1)
Other	85 (5.9)
University	
Bari	248 (17.3)
Naples	161 (11.3)
Rome	1021 (71.4)
Father‘s educational level	
*n (%)*	
Elementary	36 (2.5)
Middle school	393 (27.5)
High school	710 (49.7)
Degree	291 (20.3)
Mother‘s educational level	
*n (%)*	
Elementary	43 (3.0)
Middle school	331 (23.1)
High school	714 (49.9)
Degree	342 (23.9)
Residential status	
*n (%)*	
on-site	868 (60.7)
off-site	562 (39.3)
If off-site, spending lockdown with own family	
*n (%)*	
Yes	368 (65.5)
No	194 (34.5)
Quarantine	
*n (%)*	
Yes	27 (1.9)
No	1403 (98.1)
Doing housework	
*n (%)*	
Yes	1234 (86.3)
No	196 (13.7)
Going grocery	
*n (%)*	
Yes	720 (50.3)
No	710 (49.7)
Walking pets	
*n (%)*	
Yes	348 (24.3)
No	1082 (75.7)

**Table 2:** Changes in BMI and time spent in sedentary time and PA reported by participants during lockdown with related *p* values.

Variables *Median* ± *IQR*	*T*_0_	*T*_1_	*p* Value	*r* Value
BMI	22.6 ± 4.2	22.5 ± 4.6	0.96	0.00
Total sedentary time *min/day*	240 ± 240	480 ±300	0.00	0.78
Sleeping/napping time *min/day*	420 ± 120	480 ± 120	0.00	0.39
Total PA *min/week*	520 ± 820	270 ± 340	0.00	0.71

## Supplementary Materials

The following are available online at https://www.mdpi.com/1660-4601/17/17/6171/s1, Table S1. Results of the logistic regression model built on the achievement of recommended PA levels during lockdown as the outcome.

## References

[1] World Health Organization Coronavirus Disease 2019 Situation Reports. https://www.who.int/emergencies/diseases/novel-coronavirus-2019/situation-reports.

[2] World Health Organization (2020). 2019 Novel Coronavirus (2019-nCoV): Strategic Preparedness and Response Plan.

[3] Napoli C., Dente M.G., Kärki T., Riccardo F., Rossi P., Declich S. (2015). Network for the Control of Cross-Border Health Threats in the Mediterranean Basin and Black Sea. Screening for Infectious Diseases among Newly Arrived Migrants: Experiences and Practices in Non-EU Countries of the Mediterranean Basin and Black Sea. Int. J. Environ. Res. Pub. Health.

[4] Narici M., De Vito G., Franchi M., Paoli A., Moro T., Marcolin G., Grassi B., Baldassarre G., Zuccarelli L., Biolo G. (2020). Impact of sedentarism due to the COVID-19 home confinement on neuromuscular, cardiovascular and metabolic health: Physiological and pathophysiological implications and recommendations for physical and nutritional countermeasures. Eur. J. Sport Sci..

[5] Peçanha T., Goessler K.F., Roschel H., Gualano B. (2020). Social isolation during the COVID-19 pandemic can increase physical inactivity and the global burden of cardiovascular disease. Am. J. Physiol. Heart Circ. Physiol..

[6] Mazza C., Ricci E., Biondi S., Colasanti M., Ferracuti S., Napoli C., Roma P. (2020). A Nationwide Survey of Psychological Distress among Italian People during the COVID-19 Pandemic: Immediate Psychological Responses and Associated Factors. Int. J. Environ. Res. Pub. Health.

[7] Presidency of the Italian Council of Ministries (2020). Decreto del Presidente del Consiglio dei Ministri 8 marzo 2020. Ulteriori Disposizioni Attuative del Decreto-Legge 23 Febbraio 2020, n. 6, Recante Misure Urgenti in Materia di Contenimento e Gestione Dell’emergenza Epidemiologica da COVID-19. (20A01522). Gazzetta Ufficiale della Repubblica Italiana Serie Generale n.59 del 8 Marzo 2020.

[8] Presidency of the Italian Council of Ministries (2020). Decreto del Presidente del Consiglio dei Ministri 11 marzo 2020. Ulteriori Disposizioni Attuative del Decreto-Legge 23 Febbraio 2020, n. 6, Recante Misure Urgenti in Materia di Contenimento e Gestione Dell’emergenza Epidemiologica da COVID-19, Applicabili Sull’intero Territorio Nazionale. (20A01605). Gazzetta Ufficiale della Repubblica Italiana n.64 del 11 Marzo 2020.

[9] Tremblay M.S., Aubert S., Barnes J.D., Saunders T.J., Carson V., Latimer-Cheung A.E., Chastin S.F.M., Altenburg T.M., Chinapaw M.J.M., Terminology Consensus Project Participants (2017). Sedentary Behavior Research Network (SBRN)—Terminology Consensus Project process and outcome. Int. J. Behav. Nutr. Phys. Act..

[10] World Health Organization (2010). Global Recommendations on Physical Activity for Health.

[11] Gallè F., Sabella E.A., Da Molin G., Liguori G., Montagna M.T., Orsi G.B., Valerio G., Napoli C. (2019). A cross-sectional study investigating lifestyle and weight perception of undergraduate students in southern Italy. BMC Public Health.

[12] World Health Organization Stay Physically Active during Quarantine. http://www.euro.who.int/en/health-topics/health-emergencies/coronavirus-covid-19/novel-coronavirus-2019-ncov-technical-guidance-OLD/stay-physically-active-during-self-quarantine.

[13] Italian Ministry of Health COVID-19—Come Seguire Stili di Vita Corretti. http://www.salute.gov.it/portale/nuovocoronavirus/dettaglioContenutiNuovoCoronavirus.jsp?lingua=italiano&id=5392&area=nuovoCoronavirus&menu=vuoto.

[14] Istituto Superiore di Sanità Manteniamoci Attivi, Anche a Casa. https://www.epicentro.iss.it/coronavirus/sars-cov-2-stili-vita-attivita-fisica.

[15] Zhong B.L., Luo W., Li H.M., Zhang Q.Q., Liu X.G., Li W.T., Li Y. (2020). Knowledge, attitudes, and practices towards COVID-19 among Chinese residents during the rapid rise period of the COVID-19 outbreak: A quick online cross-sectional survey. Int. J. Biol. Sci..

[16] Gallè F., Sabella E.A., Da Molin G., De Giglio O., Caggiano G., Di Onofrio V., Ferracuti S., Montagna M.T., Liguori G., Orsi G.B. (2020). Understanding Knowledge and Behaviors Related to CoViD-19 Epidemic in Italian Undergraduate Students: The EPICO Study. Int. J. Environ. Res. Pub. Health.

[17] Taghrir M.H., Borazjani R., Shiraly R. (2020). COVID-19 and Iranian Medical Students; A Survey on Their Related-Knowledge, Preventive Behaviors and Risk Perception. Arch. Iran. Med..

[18] Chu A., Ng S., Koh D., Müller-Riemenschneider F. (2018). Domain-Specific Adult Sedentary Behaviour Questionnaire (ASBQ) and the GPAQ Single-Item Question: A Reliability and Validity Study in an Asian Population. Int. J. Environ. Res. Pub. Health.

[19] Craig C.L., Marshall A.L., Sjöström M., Bauman A.E., Booth M.L., Ainsworth B.E., Pratt M., Ekelund U., Yngve A., Sallis J.F. (2003). International physical activity questionnaire: 12-country reliability and validity. Med. Sci. Sports Exerc..

[20] Rosenberg D.E., Norman G.J., Wagner N., Patrick K., Calfas K.J., Sallis J.F. (2010). Reliability and validity of the Sedentary Behavior Questionnaire (SBQ) for adults. J. Phys. Act. Health.

[21] Lynch B., Friedenreich C., Khandwala F., Liu A., Nicholas J., Csizmadi I. (2014). Development and testing of a past year measure of sedentary behavior: The Sit-Q. BMC Pub. Health.

[22] Wijndaele K., De Bourdeaudhuij I., Godino J.G., Lynch B.M., Griffin S.J., Westgate K., Brage S. (2014). Reliability and validity of a domain-specific last 7-d sedentary time questionnaire. Med. Sci. Sports Exerc..

[23] Nunnally J.C. (1978). Psychometric Theory.

[24] Kline P. (1999). The Handbook of Psychological Testing.

[25] Cohen J. (1988). Statistical Power Analysis for the Behavioral Sciences.

[26] Huckins J.F., daSilva A.W., Wang W., Hedlund E., Rogers C., Nepal S.K., Wu J., Obuchi M., Murphy E.I., Meyer M.L. (2020). Mental Health and Behavior of College Students During the Early Phases of the COVID-19 Pandemic: Longitudinal Smartphone and Ecological Momentary Assessment Study. J. Med. Internet Res..

[27] Deschasaux-Tanguy M., Druesne-Pecollo N., Esseddik Y., Szabo de Edelenyi F., Alles B., Andreeva V.A., Baudry J., Charreire H., Deschamps V., Egnell M. (2020). Diet and physical activity during the COVID-19 lockdown period (March–May 2020): Results from the French NutriNet-Sante cohort study. medRxiv.

[28] Constandt B., Thibaut E., De Bosscher V., Scheerder J., Ricour M., Willem A. (2020). Exercising in Times of Lockdown: An Analysis of the Impact of COVID-19 on Levels and Patterns of Exercise among Adults in Belgium. Int. J. Env. Res. Public Health.

[29] Cheval B., Sivaramakrishnan H., Maltagliati S., Fessler L., Forestier C., Sarrazin P., Orsholits D., Chalabaev A., Sander D., Ntoumanis N. Relationships Between Changes in Self-Reported Physical Activity and Sedentary Behaviours and Health During the Coronavirus (COVID-19) Pandemic in France and Switzerland. https://osf.io/preprints/sportrxiv/ydv84.

[30] Kantomaa M.T., Tammelin T.H., Näyhä S., Taanila A.M. (2007). Adolescents’ physical activity in relation to family income and parents’ education. Prev. Med..

[31] Amireault S., Godin G., Vézina-Im L.A. (2013). Determinants of physical activity maintenance: A systematic review and meta-analyses. Health Psychol. Rev..

[32] Cellini N., Canale N., Mioni G., Costa S. (2020). Changes in sleep pattern, sense of time and digital media use during COVID-19 lockdown in Italy. J. Sleep Res..

[33] Maselli M., Ward P.B., Gobbi E., Carraro A. (2018). Promoting Physical Activity among University Students: A Systematic Review of Controlled Trials. Am. J. Health Promot..

