# Supplementary material for: Sedentary Behaviors and Physical Activity of Italian Undergraduate Students during Lockdown at the Time of CoViD−19 Pandemic
Source: Int J Environ Res Public Health. 2020 Aug 25;17(17):6171. doi: 10.3390/ijerph17176171 (PMC7504707; doi:10.3390/ijerph17176171)
Supplement: Supplementary file 1 [file ijerph-17-06171-s001.pdf]

Supplementary Table 1. Results of the logistic regression model built on the achievement of recommended PA levels during lockdown as the outcome.

**Independent Variable**	**Achieving recommended PA level OR (CI95%)**
Age	
≤ 21 years	*Reference*
≥ 22 years	0.78 (0.61–0.98)*
Gender	
Male	*Reference*
Female	1.30 (1.01-1.65)*
University	
Bari	*Reference*
Naples	1.97 (1.45-2.67)**
Rome	1.68 (1.09-2.61)*
Parents' educational level	
Not graduated	*Reference*
Graduated	1.31 (1.03–1.68)*
Previous BMI	
Under/Normal weight	*Reference*
Overweight/Obese	1.03 (0.78-1.36)
Previous achievement of recommended PA level	
No	*Reference*
Yes	4.53 (3.59–5.72)**

OR (CI95%): Odds Ratio (95% Confidence Interval); *p<0.05; ** p<0.01.
